# Sustainability in breast surgery: a narrative review and guide for change

**DOI:** 10.1308/rcsann.2025.0036

**Published:** 2025-06-19

**Authors:** J Worsfold, S Saha

**Affiliations:** East Suffolk and North Essex NHS Foundation Trust, UK

**Keywords:** Sustainability, Breast, Surgery, Narrative, Review

## Abstract

**Introduction:**

The purpose of this review was to evaluate sustainable interventions in breast surgery that reduce carbon dioxide equivalent (CO_2_e) emissions and financial costs, supporting the National Health Service (NHS) goal of net zero carbon emissions by 2040.

**Methods:**

A literature search was conducted using MEDLINE via PubMed up to 2024, targeting studies on sustainability in breast surgery. Search terms included sustainability-related and breast surgery-specific keywords. Owing to limited evidence, a narrative review was performed, incorporating findings from published literature, local audits and environmental impact assessments. Interventions were analysed across the perioperative pathway.

**Findings:**

Only one study specifically addressed sustainability in breast surgery, highlighting a significant evidence gap. However, multiple practical interventions were identified. The use of total intravenous anaesthesia reduced CO_2_e emissions by more than 90% compared with inhalational agents. Reusable surgical gowns and drapes offered a lower CO_2_e (0.44kg vs 0.77kg per gown) and were cost effective. Switching from intravenous to oral paracetamol saved 47kg CO_2_e annually and reduced costs (£0.02 vs £0.49 per dose). Other sustainable practices included waste segregation, minimising unused equipment and implementing electronic documentation. Local audits revealed that 7.5% of opened sutures were unused, highlighting opportunities to reduce waste.

**Conclusions:**

Simple, sustainable changes in breast surgery can significantly reduce both environmental and financial costs. Despite a lack of robust data, practical measures already in use show clear benefits. Future research should focus on whole lifecycle analyses of sustainable interventions, providing evidence to support sustainable practices. Incorporating these interventions across surgical disciplines can meaningfully contribute to achieving NHS net zero goals.

## Introduction

The National Health Service (NHS) has set the goal of achieving net zero by 2040 with the aim of achieving an 80% reduction by 2032.^1^ The health and social care system in England is responsible for 45% of the United Kingdom’s (UK) carbon footprint.^2^ Surgery is one of the areas contributing to the carbon footprint and the contribution is multifaceted. We aim to explore each of these areas in detail, in line with the green theatre checklist, with a focus on breast surgery practice.^3^

To understand the impact of sustainable interventions we compare the carbon cost of both conventional equipment and sustainable alternatives. These are measured according to the carbon dioxide equivalent (CO_2_e), a way of displaying the carbon cost of an intervention when compared with the emission of CO_2_ as a greenhouse gas. To provide some context, we can all understand that a train journey has fewer emissions than a domestic flight. A domestic flight has a CO_2_e of 246g/km. So a flight from London to Edinburgh would have a CO_2_e of 160kg. The same journey by train has a CO_2_e of 24kg.^4^ When comparing reusable medical equipment with disposable, we need to take into consideration the lifecycle of the equipment and the CO_2_ costs of its maintenance and sterilisation, to make this comparison accurate.

Breast surgery is important from a sustainability perspective given the high incidence of 56,000 new diagnoses of breast disease per year.^5^ Some 80% of patients diagnosed with breast cancer will have surgical treatment at some stage in their pathway.^6^

This review focuses on patients with breast disease undergoing breast surgery procedures. The focus is on the intervention of sustainable practice. This is compared with non-sustainable or disposable equipment use. The outcomes assessed include CO_2_e and the financial cost of the interventions. Although not independently assessed, an awareness of the impact of landfill waste is also considered when considering the value of reusable equipment compared with single-use disposable alternatives.

Although the interventions and considerations are presented in the context of breast surgery practice, many of the principles and outcomes can be utilised across the full range of surgical specialties. Any member of the surgical team from any surgical sub-specialty can take the interventions and ideas outlined forwards, as we move together towards a more sustainable future.

## Methods

A comprehensive literature search was conducted to identify studies evaluating sustainable practices in breast surgery. We searched the MEDLINE database via PubMed from inception to 2024 using a combination of Medical Subject Headings and free-text terms. The search strategy included terms related to sustainability (e.g. ‘sustainability’, ‘green surgery’, ‘environmental impact’, ‘carbon footprint’, ‘waste reduction’, ‘energy efficiency’ and ‘recycling’) and breast surgery (e.g. ‘breast surgery’, ‘mastectomy’, ‘lumpectomy’, ‘breast reconstruction’ and ‘oncoplastic surgery’). To capture relevant study designs, we included terms for randomised controlled trials, clinical trials, cohort studies, systematic reviews and comparative studies.

This search yielded only one study, which speaks volumes to the current lack of evidence base for sustainable practice in breast surgery. Much more research is required to understand the financial and CO_2_ costs of individual equipment and interventions used on the day of surgery.

Because of the limited available evidence specifically targeted at sustainable practice in breast surgery, we proceeded to conduct a narrative review of current sustainable interventions options. We hope this will prompt further discussion and research into the specific environmental and financial benefits of sustainable surgical practice. These have been subdivided into the different elements of a patient’s journey on the day of surgery.

## Findings

### Anaesthesia

The impact of anaesthesia on climate change has been extensively studied. There is a need to move away from traditional inhaled anaesthetic agents because of their greenhouse gas effect. Over time, anaesthetic gases from surgery have contributed 200kt CO_2_e in emissions.^7^ There is variation in the CO_2_e between inhaled anaesthetic agents. Nitrous oxide and desflurane have a significantly higher CO_2_e than sevoflurane and isoflurane.^7^ Total intravenous anaesthesia (TIVA) is used regularly in our unit to reduce the CO_2_e associated with the anaesthetic. A study comparing TIVA with a mixed inhalational anaesthetic found the carbon footprint per intervention with TIVA to be 2.42kg CO_2_e vs 48.85kg CO_2_e in the mixed group.^8^ A shift towards TIVA has a clear benefit from a CO_2_ emissions perspective. In addition there is evidence of reduced postoperative nausea and vomiting with TIVA use.^9^ It has also been found that TIVA has potential long-term survival benefits when used in cancer surgery compared with inhalational anaesthesia.^10^

Oral pre-medications as opposed to intravenous (IV) use also allowed us to reduce the carbon footprint and cost associated with medications used during the anaesthetic. Switching to preoperative oral paracetamol allowed us to save 47kg CO_2_e annually. The cost of a dose of oral paracetamol is £0.02 compared with £0.49 for IV paracetamol.^11^ This is a significant saving when considering the volume of cases this can apply to. In addition to this, we encourage oral pre-loading of fluids up to 1h preoperatively. This is cheaper and more sustainable than routine IV fluids in the perioperative setting. It can also help to reduce the number of plastic syringes and giving sets required for an individual case.^12^

Paperless anaesthetic charts and records further reduce the emissions associated with the printing and production of paper notes.^13^ This is something we aim to achieve across all domains, including in preoperative clinics, together with a move towards electronic consent forms.

### Preparing for surgery

Reusable surgical gowns are associated with a 64% reduction in natural resource energy consumption compared with disposable gowns.^14^ This includes the expenditure required to wash and sterilise the reusable gowns. The financial cost of a reusable gown is £2.15 per use compared with £2.20 for a disposable gown. The CO_2_e for a reusable gown is 0.44kg compared with 0.77kg for the disposable gown.^15^ This is one example in which both cost and CO_2_e are reduced with the use of reusable equipment and provides a strong recommendation for their use and implementation for all units across all surgical specialties.

Other examples of reusable equipment include surgical scrub hats, surgical drapes and trolley covers. Utilising reusable alternatives can reduce landfill waste and CO_2_ emissions, and often proves to be more cost effective.^16^

There is evidence that a full surgical scrub may not be required between each case in order to reduce surgical site infection. A sustainable option to consider is an alcohol scrub only for subsequent cases during an operating list. This is associated with a reduction in water consumption and energy costs compared with a conventional scrub before every case on a list.^17^

Additional sustainable changes can be made through the rationalisation of equipment and medications used in the perioperative period for each patient. Considerations include the use of preoperative antibiotic prophylaxis and the use of devices; for example, catheters and pneumatic anti-embolism devices. These should be tailored to individual needs and not simply used routinely in all patients.

The requirement for preoperative blood tests, including group and save samples, should be rationalised to avoid the unnecessary cost and CO_2_e associated with these tests. Local guidelines filtered through to the anaesthetic preassessment team are utilised to prevent unnecessary preoperative blood tests being carried out. These decisions can be adjusted on a case-by-case basis to ensure patient safety and maximise sustainability where appropriate.^3^

### Intraoperative equipment

During the operation only essential equipment should be opened for each case. By keeping instruments and equipment such as drains and sutures on standby if it is unclear whether they will be required, we can reduce unnecessary wastage. An audit locally highlighted that 7.5% of sutures opened were not actually used during the case. This audit was conducted in June 2023 at the Colchester Hospital site in patients undergoing elective breast surgery over a 1-month period.^18^

Engagement with local sterile services is essential to ensure they have capacity to sterilise reusable equipment between cases. This extends to kidney dishes, gallipots and irrigation fluid jugs. In addition, rationalising surgical sets to contain only case-specific essential items avoids unused equipment being recycled through the sterilisation process.

Single-use surgical clip applicators can be replaced with cartridges of clips and a reusable clip applicator. This reduces both the waste and CO_2_ costs associated with the use of disposable applicators.^19^

Reusable light handles are available in most theatres, which again help to reduce wastage compared with disposable handles. Surgical preparations applied with a gallipot as opposed to the use of chlorhexidine prepackaged in disposable plastic will also reduce carbon costs and landfill wastage.

Patient warming is another area in which changes can be made to improve sustainability. Forced air warming devices (e.g. Bair Hugger) use a disposable component and have a significant electricity consumption to heat the air. An alternative is the use of electric patient-warming devices such as Hot Dog. The Hot Dog is a system comprising two reusable electric blankets that are placed beneath and on top of the patient. This has several benefits in terms of emissions through reduced electricity usage and the associated financial cost. It also allows the patient to be heated efficiently from underneath without disturbing the operating field. There is also no disposable or single-use component.^20^

### After the operation

The measures taken to reduce the emissions associated with surgery do not stop at the end of the operating list. Where reusable equipment is not available, recycling is essential to reduce landfill waste and improve the sustainable disposal of equipment. We have set up waste segregation in the operating theatre to maximise the opportunity to recycle paper and plastics that would otherwise be disposed of in the general waste.^21^ The importance of waste segregation goes beyond recycling. It is imperative that waste, if not recyclable, is segregated into the correct bin. Disposal of sharps and clinical waste has a four times greater cost than the disposal of general waste.^22^

Consideration should also be given to the use of technetium (Tc) and the procedures surrounding radioactive waste storage and disposal. Ensuring that waste is separated appropriately will reduce the costs associated with clinical waste and allow for increased recycling. Following appropriate quarantine of Tc-99 waste can be recycled as normal.

There are alternatives to Tc for dual sentinel node localisation. Some of these are non-radioactive; for example, Magtrace. Magtrace can be injected at the time of a clinic visit up to 30 days preoperatively and is associated with reduced financial costs compared with Tc.^23^ A reduction in the number of clinic visits for patients travelling for injections on the day before surgery can reduce CO_2_ associated with additional attendance. There are no current data evaluating the specific CO_2_e of Magtrace vs Tc.

With the increased use of reusable equipment there need to be measures in place to ensure their appropriate repair and maintenance. This will prolong the lifespan of reusable equipment and further reduce the CO_2_e and financial costs compared with single-use alternatives. There may be higher upfront financial costs, however, and ongoing maintenance costs. Lifecycle analysis of both costs and CO_2_e is crucial to make a clear comparison.

Other simple measures to reduce cost and emissions include ensuring a full electronic shutdown and switching off the equipment in the theatre at the end of the list. This is a simple way to reduce potential electricity costs and associated emissions. A study that looked at a large range of equipment estimated that 44,774kg CO_2_e per year could be saved by switching off equipment at the end of elective lists and over the weekend when not in use. There was also an associated financial saving, which one health board estimated to be £26,000 per year.^24^

## Discussion

From the initial literature review and search it is clear that a lot more research is required to enable a full assessment of the benefits of sustainable interventions for patients undergoing breast surgery. It is easy to ignore sustainability while immersed in a busy surgical service. This review aims to bring to the fore the potential interventions that can result in sustainable changes, have benefits in terms of financial cost and reduce the amount of waste we send to landfill.

Future research should focus on analysis of the specific CO_2_ and financial costs of sustainable and non-sustainable resources in the operating theatre, in particular looking at whole lifecycle analysis of equipment to make a fair comparison. By understanding these parameters we can drive change towards a more sustainable future in breast surgery and beyond.

## Conclusions

The main takeaway from this review is that simple measures can make a big difference. I want you to consider sustainability the next time you walk into your operating theatre. Can you make use of reusable equipment such as gowns and drapes? Look at the waste produced at the end of your surgery, is there any facility for recycling available? Given the net zero aims of the NHS, there is often significant managerial support for sustainable projects and initiatives. These changes are often not only sustainable, but also reduce costs over the longer term compared with single-use disposable alternatives. It is easy to maintain the status quo; however, as demonstrated, simple basic interventions can make a big difference, especially when multiplied across a variety of hospitals and surgical specialties. Consider the number of disposable gowns that are sent to landfill every year. The scale of single-use items and waste produced by the NHS is substantial. Every individual has a role to play in creating a sustainable NHS and future. The authors welcome any correspondence to help facilitate further collaboration towards increased sustainability. Step by step we can create a sustainable surgical service which will benefit patients and staff for years to come.
